# Early social adversity modulates the relation between attention biases and socioemotional behaviour in juvenile macaques

**DOI:** 10.1038/s41598-021-00620-z

**Published:** 2021-11-04

**Authors:** Holly Rayson, Alice Massera, Mauro Belluardo, Suliann Ben Hamed, Pier Francesco Ferrari

**Affiliations:** 1 Institut des Sciences Cognitives Marc Jeannerod, Centre National de la Recherche Scientifique, Université Claude Bernard Lyon 1, Bron, France; 2 Unit of Neuroscience, Department of Medicine and Surgery, University of Parma, Parma, Italy

**Keywords:** Psychology, Animal behaviour

## Abstract

Affect-biased attention may play a fundamental role in early socioemotional development, but factors influencing its emergence and associations with typical versus pathological outcomes remain unclear. Here, we adopted a nonhuman primate model of early social adversity (ESA) to: (1) establish whether juvenile, pre-adolescent macaques demonstrate attention biases to both threatening and reward-related dynamic facial gestures; (2) examine the effects of early social experience on such biases; and (3) investigate how this relation may be linked to socioemotional behaviour. Two groups of juvenile macaques (ESA exposed and non-ESA exposed) were presented with pairs of dynamic facial gestures comprising two conditions: neutral-threat and neutral-lipsmacking. Attention biases to threat and lipsmacking were calculated as the proportion of gaze to the affective versus neutral gesture. Measures of anxiety and social engagement were also acquired from videos of the subjects in their everyday social environment. Results revealed that while both groups demonstrated an attention bias towards threatening facial gestures, a greater bias linked to anxiety was demonstrated by the ESA group only. Only the non-ESA group demonstrated a significant attention bias towards lipsmacking, and the degree of this positive bias was related to duration and frequency of social engagement in this group. These findings offer important insights into the effects of early social experience on affect-biased attention and related socioemotional behaviour in nonhuman primates, and demonstrate the utility of this model for future investigations into the neural and learning mechanisms underlying this relationship across development.

## Introduction

In the face of limited perceptual and cognitive resources, attention mechanisms enable the brain to manage competing demands in the everyday environment by prioritizing a subset of stimuli for dedicated processing. Such mechanisms guide behaviour from the earliest months postpartum, serving as a fundamental base for learning, self-regulation, and memory^1,2^. *Affect-biased* attention specifically is posited to play a broad and pervasive role in early socioemotional development^3,4^, with emerging affect biases shaping an infant’s experience of their environment via preferential processing of threat- and reward-related information. This, in turn, is thought to support the emergence of adaptive approach and avoidance behaviour^5,6^. However, specific affect-biases have also been linked to poor socioemotional functioning later on in development (e.g.^7,8^), and many questions remain concerning the mechanisms through which affect-biased attention arises and may relate to both typical and pathological outcomes.

Biased attention towards threat-relevant information serves an essential survival function^9^. It is unsurprising, therefore, that most affect-bias studies have focused on threatening stimuli such as angry or fearful versus neutral faces. An attention bias to threat (ABT) emerges during the first year postpartum^5,10^, and has been linked to positive socioemotional outcomes in the form of secure infant attachment^6^. Conversely, ABT has also been associated with emotion regulation difficulties, social withdrawal, and anxiety in both adults and younger populations^11,12^, with cognitive models of anxiety attributing a causal relation to ABT in the development and/or maintenance of anxiety^13–15^. This apparent contradiction suggests that any increased vulnerability conferred by threat-biased attention may arise from an early-emerging, normative threat bias^16,17^, with excessive ABT or a failure to inhibit ABT exacerbating the risk for psychopathology^18^. Inconsistent findings concerning a link between ABT and anxiety in childhood, with no relation often found despite ABT presence (e.g.^17^), is in keeping with this idea, but so far, very little is known about how ABT and socioemotional function interact across development.

An attention bias to positive stimuli (ABP) such as happy faces may also play an important role in early socioemotional functioning. Such positive biases are often conceptualized as a bias towards rewarding stimuli^3^. Relatively little is known about the emergence of ABP, but evidence suggests this also arises at an early stage in development^10,19^. ABP has been associated with several aspects of positive socioemotional functioning in children and adults, including social engagement and prosocial behaviour^20^, adaptive emotion regulation skills^21^, and positive affect^22^. Notably, ABP has also been linked to lower levels of anxiety (e.g.^23^), and may act as a protective factor in developmental populations at increased risk for poor socioemotional outcomes. This includes risk for anxiety and internalizing problems in behaviourally inhibited and previously institutionalized children^20,24–26^. Nevertheless, findings linking ABP to anxiety in younger populations are again mixed, with some studies failing to find any relation between ABP and anxiety in childhood^8,27^.

Nonhuman primate (NHP) models could add significantly to our understanding of affect-biased attention and its role in early socioemotional functioning. Adopting a comparative developmental approach can provide unique insights into the origins of human cognition, highlighting similarities and divergences in our evolutionary history^28,29^. However, it is currently unclear whether affect biases comparable to those in humans are present in early NHP development, and how these may relate to other specific developmental outcomes. Rhesus macaque (*Macaca mulatta*) monkeys are very similar to humans in terms of cognition, socio-affective characteristics, and brain organization, and are thus commonly utilized to investigate the aetiology of various psychiatric and neurodevelopmental disorders, including anxiety (see^30,31^). Macaques also live in large social groups, have an extended period of development comprising distinct infant, juvenile (pre-adolescent and adolescent), and adult periods, and the early macaque mother-infant relationship shares many commonalities with humans^32^. The macaque model is, therefore, especially well-suited to developmental studies, and could provide particularly valuable information concerning the mechanisms underlying ABT and ABP emergence. Accordingly, we adopted a macaque model in the current study with the goal of furthering our understanding of affect-biased attention in NHPs, and investigating the suitability of this as a translational model of its development and related socioemotional outcomes.

Early-emerging ABT also appears normative in macaques, assessed via presentation of threatening versus neutral faces^29,33,34^. To our knowledge, only one macaque study has investigated ABP specifically, with no bias towards a positive, affiliative facial gesture (‘lipsmacking’) versus a neutral face revealed at any stage in development^29^. Nevertheless, this lack of bias may have resulted from difficulty in discriminating between the two static images, as lipsmacking, a highly rhythmic and dynamic facial gesture, is very difficult to portray in a static stimulus. To date, the majority of both human and NHP studies that have utilized paired affective versus neutral facial stimuli to investigate affect-biased attention have used static images. The use of dynamic facial stimuli, however, improves various aspects of perception and enhances attention biases in human adults^35–37^, and recruits dissociable neural pathways from those involved in the perception of static faces^38,39^. This issue of static versus moving faces is especially pertinent for developmental research. Dynamic stimuli can enhance neural and behavioural discrimination of emotional versus neutral faces from early infancy^40,41^, and attention to emotional expressions is modulated by stimulus motion across childhood, adolescence, and into adulthood^42^. Altogether, this highlights the importance of adopting more ecologically valid, dynamic facial stimuli in studies of affect-biased attention, and indeed, there is a growing movement within the wider research community towards the use of more naturalistic stimuli in studies of social attention with humans and NHPs (e.g.^43^).

To address outstanding questions concerning the mechanisms through which affect-biased attention emerges and relates to both typical and atypical functioning, it is critical to consider which factors may contribute to individual differences in ABT and ABP, and how such differences may confer vulnerability or resilience. It is well established that early social adversity can increase risk for a number of adverse socioemotional outcomes, including anxiety and reduced social engagement in humans and macaques (e.g.^44–47^). A small number of studies have linked elevated ABT specifically to early social deprivation^20,25^ and maternal anxiety^10^ in children and human infants, with maternal abuse and over-protectiveness associated with ABT magnitude in infant and adolescent macaques^33,34^. Early social deprivation may also impact ABP, with ‘care-as-usual’ versus foster home placement related to a reduced or absent bias towards happy faces, and greater ABP to more social engagement and fewer internalizing problems^20,25^ in the context of early institutionalization. It remains unknown whether early social adversity has similar effects on the relation between affect-biased attention and comparable socioemotional outcomes in NHPs.

The current study was designed to investigate both ABT and ABP in pre-adolescent macaques using dynamic facial stimuli, and to examine whether such biases are linked to socioemotional functioning; specifically, anxiety-like behaviour and social engagement. To consider the effects of early social adversity, we assessed two groups of juvenile macaques (aged 2.5 years), one mother-reared and one peer-reared. Peer-rearing is often adopted in macaque models of early social adversity, and has been associated with both increased anxiety and decreased social behaviour^45,47^. Our hypotheses were: (1) although all animals will demonstrate attention biases, peer-reared animals will demonstrate greater ABT, and mother-reared animals will demonstrate greater ABP; (2) greater ABT will be related to more anxiety-like behaviour, but greater ABP will be related to less; and (3) greater ABP will be related to more social engagement.

## Methods

### Subjects and housing conditions

The sample consisted of 21 juvenile rhesus macaques (*Macaca mulatta*), 11 mother-reared (six female) and 10 peer-reared (5 female). Subjects were aged around 2.5 years at the time of this study (mother-reared; M = 943.18 days, SD = 17.98: peer-reared; M = 956.2 days, SD = 20.24). Subjects were housed at the *Institut des Sciences Cognitives Marc Jeannerod, CNRS*, in mixed mother- and peer-reared social groups of 5–6 animals. All housing and procedures conformed to current guidelines concerning the care and use of laboratory animals (European Community Council Directive No. 86-609), and were approved by our local ethics board, ‘Comité d’Ethique Lyonnais pour les Neurosciences Expérimentales’ (CELYNE) C2EA #42 (03.10.18), and the French Ministry of Research (10.10.18); project reference APAFIS#15091_2018071014483295_v2. All reporting here conforms to the recommendations in the ARRIVE Guidelines for Reporting Animal Research.

All subjects were born and raised at the *Laboratory of Comparative Ethology at the National Institutes of Health, US*. Peer-reared animals were raised in a nursery with access to same-aged peers; see^48^ for more rearing protocol details. Rearing procedures were approved by the NICHD and the University of Maryland Animal Care and Use Committee, and adhered to the NIH Guide for the Care and Use of Laboratory Animals. Animals were relocated to the *Rousset Primatological Station, CNRS, France* at two years of age. More information about the rearing protocol and housing can be found in the SI.

### Facial gesture stimuli

Stimuli for the attention bias task consisted of short, dynamic video clips (5 s) of an unfamiliar adult female macaque performing three types of facial movements: (1) neutral facial movements (i.e. no gesture but with small movements of, for example, the nose and mouth); (2) lipsmacking (LPS), comprised of the rapid, rhythmic opening and closing of the mouth and pursing of the lips; and (3) open-mouth threat, comprised of the wide opening of the mouth and lowering of the jaw, with lips held in a tense position covering the teeth. The onset and duration of movement, size, brightness, contrast, spatial frequency, and overall motion levels were controlled for (see SI) to ensure that neutral, lipsmacking, and threat video stimuli did not differ in terms of low-level visual features. All videos started with a 500 ms static period showing the first frame of the video (with a neutral facial expression), followed by 4500 ms of movement (i.e. two consecutive instances of each gesture or neutral movement sequence).

### Attention bias task

Each subject was temporarily separated from their social group and placed into the testing area in another section of the room; 87 × 100 × 120 cm enclosure with a clear panelled front. Before commencing the task, a widescreen computer monitor (35 × 61 cm; 2560 × 1440 resolution) was placed 60 cm from the front of the enclosure, and animals were given five minutes to habituate to the enclosure once separated. Note, all animals had already been well familiarized with this process of separation into the testing enclosure and presentation of non-social video stimuli before the day of assessment. Animals were recorded during the task using a webcam (30 fps) placed on the top-centre of the monitor.

**Figure 1:**
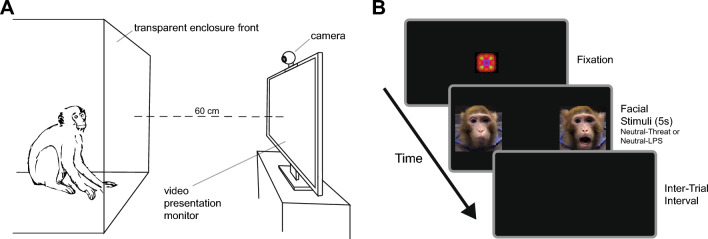
(**A**) Schematic illustration of the attention bias task set-up. (**B**) Illustration of a trial in the attention bias task. An experimenter triggered the appearance of fixation and facial stimuli screens when the subject looked towards the monitor.

Animals were presented with pairs of neutral-affective gesture stimuli comprising two conditions: (1) Neutral-Threat (five trials per subject); and (2) Neutral-LPS (five trials per subject), i.e. the positive or reward condition. Video pairs were presented for 5 s per trial, with condition order and position (left or right) of neutral-affective gesture videos counterbalanced across subjects. Before the stimuli appeared, a moving geometric pattern accompanied by a non-social sound was presented in the centre of the screen to attract the subject’s attention, with stimuli presentation then triggered by an experimenter watching the animal live on a separate monitor (not in view of the subject). Additionally, a calibration procedure was conducted before presentation of experimental stimuli, whereby images of objects (e.g. ball, toy car) were presented on the right, centre, and left of the screen. Each image was jittered up and down slightly to attract attention and was accompanied by a non-social sound. Psychopy v1.90.2^49^ was used for stimulus (calibration and experimental) presentation, with video recording onset and offset automatically triggered at the start and end of each presentation. This sequence and the experimental set-up is illustrated in Fig. 1.

Subjects’ gaze (left, right, other, offscreen) was manually coded offline, frame-by-frame, by a researcher blind to the condition being presented and the position of the neutral-gesture stimuli. A random 15% of videos were coded by a second researcher to establish reliability, with very good reliability scores obtained (*ĸ* = 0.84).

### Behavioural observation

Video recordings of the social group (50 fps) were made two times per week, once in the morning and once in the afternoon, for three weeks. Two cameras were used to capture the whole home enclosure and were synchronized offline for the manual coding of behaviour. Each animal was coded second-by-second for five minutes per recording session, totalling 30 min per animal. One experimenter coded all of the videos, with a second experimenter coding a random 15% to establish reliability (*ĸ* = 0.93).The following behaviours were coded using the focal sampling method: (a) self-scratching, self-grooming, yawns, and body shakes (i.e. behaviours reflecting anxiety in macaques; see^30^); and (b) social grooming (both give and receive), which is a primary means by which macaques maintain and strengthen social relationships^50^.

### Data analysis

Preprocessing and analyses pipeline scripts are available on GitHub (https://github.com/hrayson/attentionbiasmacaque). To calculate attention bias to threat (ABT), the proportion of time looking at the neutral and threat stimuli (out of total time looking onscreen) was calculated separately for each Neutral-Threat trial, with the neutral proportion then subtracted from the threat proportion. The equivalent approach was used to calculate attention bias to LPS stimuli, i.e. positive stimuli (ABP). A linear mixed model was then utilized to investigate potential differences between rearing groups and attention bias type at the trial level, with group (mother-reared or peer-reared), condition (threat or LPS), and their interaction included as fixed effects, and subject-specific intercepts as a random effect. Social rank (randomized Elo-ratings) was also included as a covariate, and z-scored for analysis. Model residuals were checked for normality and homogeneity. Before analysis, trials where animals failed to look at the screen during facial gesture presentation or were 2.5 SDs above or below the mean were excluded. More details about rank calculation and trial exclusion can be found in the SI.

The following behavioural indices were computed based on the behavioural observation coding: (1) *anxiety frequency*, obtained by summing occurrences of self-scratching, self-grooming, yawns, and body shakes; and (2) both *duration and frequency of social engagement*, obtained by calculating total time spent in social grooming interactions and the frequency of social grooming interactions, respectively. To explore the relation between attention biases and anxiety or social engagement at the observation session level, generalized linear mixed models were run separately for average ABT and ABP per subject, with negative binomial error distribution and a log link function. This was done with either *frequency* or *duration* of behaviour as the outcome variable. Group (mother-reared or peer-reared), ABT or ABP, and their interaction were included as fixed effects, rank (randomized Elo-ratings) as a covariate, and subject-specific intercepts as a random effect. Elo-ratings and attention biases were z-scored for analysis.

R v3.6.3^51^ was utilized to conduct these analyses (see SI for package information). *P*-values for fixed effects and interactions were obtained using Type III *F* tests for linear models, and Type III Wald χ^2^ tests for generalized linear models. Significant interactions between factors were followed up by planned pairwise comparisons of estimated marginal means which were Tukey-corrected for multiple comparisons. Significant interactions between factors and continuous variables (i.e. ABT or ABP) were followed up by planned comparison of the estimated marginal means of the linear trends of the continuous variable to 0 at each level of the factor. Effect sizes are reported as unstandardized model parameter estimates (in the scale of the model response variable). All animals were included in these analyses (n = 21; 11 mother-reared, 10 peer-reared). Descriptive statistics can be found in Table 1.

**Table 1:** Gaze measures and socioemotional behaviours.

Group	Mother-reared	Peer-reared
*Gaze measures*		
Proportion onscreen	0.60 (0.15)	0.44 (0.19)
Proportion threat (Neutral-Threat trials)	0.455 (0.1)	0.536 (0.17)
Proportion neutral (Neutral-Threat trials)	0.264 (0.103)	0.145 (0.065)
Proportion LPS (Neutral-LPS trials)	0.456 (0.094)	0.324 (0.1)
Proportion neutral (Neutral-LPS trials)	0.225 (0.069)	0.292 (0.133)
Attention bias to threat (ABT)	0.19 (0.164)	0.386 (0.151)
Attention bias to LPS (ABP)	0.231 (0.086)	0.032 (0.14)
*Socioemotional behaviour*		
Anxiety frequency	1.515 (0.701)	2.350 (1.093)
Social groom frequency	1.485 (1.599)	1.250 (0.934)
Social groom duration (s)	42.909 (45.546)	24.100 (21.869)

Proportion onscreen is the proportion (M and SD) of trial time spent attending to the screen. Proportion threat, neutral, and LPS are the proportions (M and SD) of time attending to the screen spent attending to the threat, neutral, and LPS stimuli, respectively. ABT and ABP (i.e. affective versus neutral stimuli) is the difference between proportions to threat (or LPS) and neutral in the neutral-threat, and neutral-LPS trials, respectively. Anxiety and social groom frequency are the frequencies (M and SD) of anxiety-like behaviour and social grooming during the behavioural observation period. Social groom duration (M and SD) is the total amount of time (seconds) spent in grooming interactions during the behavioural observation period.

## Results

### Attention bias to threat and LPS

**Figure 2:**
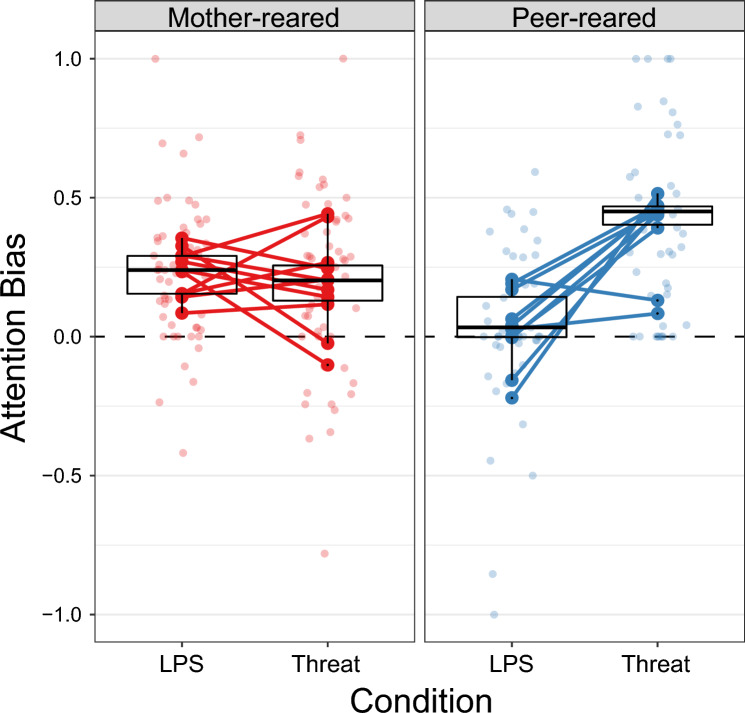
Attention biases in the Neutral-LPS and Neutral-threat conditions, for the mother-reared group (red; left) and peer-reared group (blue; right). Zero indicates no bias, positive values a bias towards LPS or threat versus neutral, and negative values a bias towards neutral versus LPS or threat. Light-coloured dots represent the bias in each trial, large dark-coloured dots indicate the average bias for each subject, and lines connect the average bias in the two conditions per subject. The median bias per group, first and third quartiles, and + or − 1.5 times the inter-quartile range from the first and third quartiles are also shown in the box plots.

First we compared the attention biases to affective stimuli in the Neutral-LPS and Neutral-Threat conditions between the mother- and peer-reared groups. This revealed a significant main effect of group [*F*(1) = 8.288, *p* = 0.007, effect size (mother-peer) = 0.036], as well as a significant group × condition interaction [*F*(1) = 22.101, *p* < 0.0001, mother-reared effect size (LPS-threat) = 0.036, peer-reared effect size (LPS-threat) =  − 0.364] (Fig. 2). Attention bias to threat (ABT) was greater in the peer-reared compared to mother-reared group [*t*(33.1) =  − 2.079, *p* = 0.045], and attention bias to LPS (i.e. positive bias; ABP) was greater in the mother-reared compared peer-reared group [*t*(34.6) = 2.879, *p* = 0.007]. In the mother-reared group, ABT and ABP were not significantly different from each other [*t*(176) = 0.628, *p* = 0.531] but in the peer-reared group, ABT was significantly greater that ABP [*t*(178) =  − 5.827, *p* < 0.0001].

One sample *t*-tests also confirmed that in the mother-reared group, ABT [*t*(10) = 3.841, *p* = 0.003] and ABP [*t*(10) = 8.905, *p* < 0.0001] were significantly different from zero, whereas in the peer-reared group, only ABT [t(9) = 8.093, *p* < 0.0001] was significantly different from zero.

### Relation between attention biases and socioemotional behaviour

**Figure 3:**
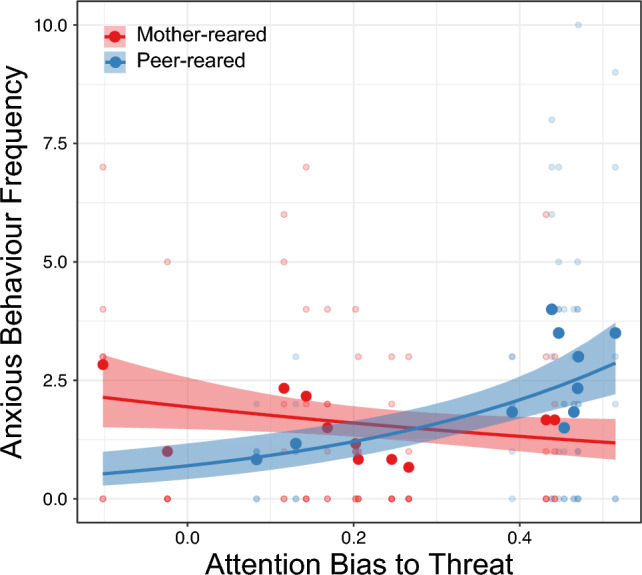
The relation between attention bias to threat (ABT) and frequency of anxiety-like behaviour in the mother-reared (red) and peer-reared (blue) group. Each light-coloured dot represents an individual behavioural observation session, large dark-coloured dots represent the subject mean, dark lines indicate the model fit, and shaded regions around the lines denote + or − SE.

Having established a difference in attention biases between groups and conditions, we then sought to determine if attention biases were related to the frequency of anxious behaviour in the two rearing groups. We found a group × ABT interaction [*χ*^2^(1) = 5.976, *p* = 0.015, mother-reared effect size =  − 0.178, peer-reared effect sized = 0.504] (Fig. 3), with greater ABT related to more frequent anxiety-like behavior in the peer-reared group [*z* = 2.256, *p* = 0.024]. There was no significant main effect of ABP or the relation between ABP and frequency of anxious behaviour in either rearing group (both *p* > 0.411).

**Figure 4:**
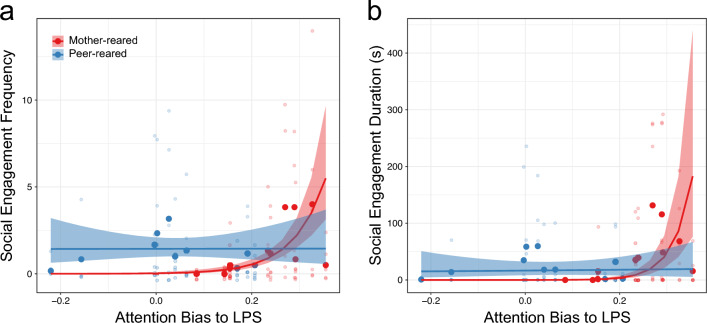
The relations between attention bias to LPS (i.e. to positive stimuli; ABP) and social engagement in the mother–reared (red) and peer-reared (blue) group. Each light-coloured dot represents an individual behavioural observation session, large dark-coloured dots represent the subject mean, dark lines indicate the model fit, and shaded regions around the lines denote + or − SE.

We then went on to investigate relations between attention biases and the frequency and duration of social engagement. There were significant main effects of ABP [*χ*^2^(1) = 10.788, *p* = 0.001], group [*χ*^2^(1) = 5.169, *p* = 0.023], and an ABP × group interaction [*χ*^2^(1) = 7.730, p = 0.005, mother-reared effect size = 2.278, peer-reared effect size = 0.004] for social engagement frequency (Fig. 4a), with greater ABP related to more frequent social engagement in the mother-reared group [*z* = 3.284, *p* = 0.001]. For social engagement duration, we found significant main effects of both ABP [*χ*^2^(1) = 16.547, *p* ≤ 0.0001] and group [*χ*^2^(1) = 5.891, *p* ≤ 0.015], and an ABP × group interaction [*χ*^2^(1) = 11.481, *p* = 0.001, mother-reared effect size = 3.784, peer-reared effect size = 0.064]; greater ABP was related to a longer duration of social engagement [*z* = 4.068, *p* < 0.0001 in the mother-reared group] (Fig. 4b). There were no significant main effects of ABT or relations between ABT and frequency or duration of social engagement (all *p* > 0.528).

## Discussion

In this study, juvenile macaques demonstrated biased attention to both threatening and affiliative dynamic facial gestures. This is in keeping with a previously demonstrated bias towards static images of open-mouth threat^29,33,34^, and provides evidence for a bias towards positive or reward-related facial gestures also. Early social deprivation was associated with the degree of bias towards both types of affective stimuli, with a greater attention bias to threat (ABT) found in peer-reared animals, and a greater attention bias to positive stimuli (ABP) found in animals reared by their mothers. Notably, ABT was also linked to anxiety-like behaviour in peer-reared animals, and ABP to levels of social engagement in mother-reared animals. These findings offer novel insights into the effects of early social adversity on affect-biased attention, and suggest that such biases could play an important role in early macaque socioemotional functioning.

The presence of a bias towards dynamic open-mouth threat in both our rearing groups provides some support for ABT emergence being part of typical macaque development. This bias was, however, greater in the peer-reared compared to mother-reared group. Therefore, although ABT emergence is likely normative in macaques^29,33^, it is possible that very early social deprivation can exacerbate this bias, even into the preadolescent juvenile period. Interestingly, such effects of early social experience on ABT are similar to those seen in human children in the context of early institutionalization^20,25^. Mother-reared animals also showed a bias towards dynamic lip-smacking (LPS) stimuli. No previous macaque study has investigated potential effects of early adversity on an attention-bias to dynamic LPS specifically, but in line with our results, there is some evidence that early rearing status can affect infants’ social responses to LPS performed by a human experimenter^52^. As a whole, the peer-reared group did not show significant ABP, again paralleling results from the available human literature concerning the effects of early social deprivation on ABP in pre-adolescence^20,25^.

Here, a greater magnitude of ABT was related to more frequent anxiety-like behaviour in the peer-reared group, suggesting that early social adversity can confer greater risk for anxiety via exaggerated or uninhibited ABT. This finding aligns with evidence suggesting that in humans, the ABT-anxiety link is not found consistently during childhood, and may be found more reliably in the context of early adverse experience. A relation between ABP and social engagement (i.e. social grooming) was also revealed in the mother-reared group, with more frequent and a longer time spent in grooming interactions associated with greater ABP. Therefore, it is possible that a bias towards reward-related stimuli also serves a positive function in macaque development. In contrast to some research with human children, we did not find a relation between greater ABP and reduced anxiety. There are a number of possible explanations for this result. For instance, some human research suggests that ABP is present in institutionalized children and linked to fewer internalizing problems only after stable fostering placement^25^. Additionally, in community samples, it may be that ABP only serves as a protective factor against anxiety in behaviourally inhibited children^53,54^.

In the case of atypical early parenting input, it is possible that uninhibited, exacerbated, or diminished affective attention biases are adaptive in the short-term, increasing offspring survival rates. Longer term, however, this may tie individuals to maladaptive trajectories of development^3,55^, increasing the risk for psychopathology and social difficulties. Reduced attention to threat has already been associated with insecure and disorganized attachment in infants^6,56^. Parental sensitivity impacts the formation of infant-caregiver attachment (e.g.^57^), with insecure and disorganized attachment implicated in the development of numerous adverse outcomes (see^58^). In the case of early social deprivation, an attachment figure is completely absent. Therefore, while it may be adaptive to avoid threat in the presence of, for example, an abusive caregiver, it may be adaptive for an infant with no social buffering to be hypervigilant towards threat. This highlights an important outstanding issue, with general versus specific mechanisms linking early adversity to negative socioemotional functioning being poorly understood^59^. Clarifying these mechanisms requires consideration of how specific types of adversity may increase risk for specific adverse outcomes. In terms of a positive bias, it may be that a lack of learning opportunities to associate positive facial gestures with reward in the absence of an attachment figure influences the emergence of a positive affect bias and related social outcomes. This idea needs to be explored more explicitly in subsequent studies, but it is in keeping with evidence for atypical reward processing in the context of early social deprivation (e.g.^60^).

Although we found a link between ABT and anxious behaviour in juvenile macaques, it remains unclear whether ABT actually played a causal or maintaining role, or simply reflected current levels of anxiety. This remains a key unanswered question in the literature. Very little research thus far has investigated how ABT relates to or predicts anxiety longitudinally, or even the developmental trajectory of ABT itself in typical or at-risk populations. There is some evidence that ABT is unstable across childhood^25,53^, with ABT links to anxiety variable across this period^53,54,61^. ABP may also vary across childhood^25,53^. Research focused on how early ABP predicts subsequent anxiety and social behaviour^17,53^ suggests that greater ABP is linked to more positive outcomes and may serve as a protective factor against anxiety from very early childhood, but again, a lack of studies measuring both ABP and socioemotional functioning at more than one time-point limits understanding of the exact role ABP serves.

Clearly, longitudinal studies assessing both affect-biased attention and socioemotional functioning at multiple time points are now needed to better understand the role of attention biases in healthy and pathological development. Such studies will be vital to determine how increased vulnerability associated with atypical ABT might arise from a normative threat bias, and how the presence or stability of affect-biased attention across development relates to positive and negative outcomes in different populations. As childhood and adolescence represent the core period of developmental risk for anxiety disorders^62^, longitudinal studies across this period are of particular importance. Examining the neural learning mechanisms through which attention biases and related outcomes arise will also be critical to address these outstanding questions, and to clarify the processes underlying these relations across different points in development. Our results suggest that the macaque model is ideal for such longitudinal research, and could add considerably to our understanding on whether universal mechanisms of development underlie affect-biased attention, and whether these evolved in primates to help offspring adapt to differences in their early social environment.

In terms of neural bases, the emergence of affect-biased attention is thought to involve attentional networks supporting alerting, orienting, and executive functions, mediated by emotion processing circuitry. This includes brain structures such as the amygdala, orbital frontal cortex (OFC), regions of prefrontal cortex, and anterior cingulate cortex^3,4^, but evidence from developmental populations is severely lacking. The amygdala and related circuitry may play a particularly crucial role in the processes underlying a relation between early adversity and affect-biases, with a wealth of evidence from the animal and human literature linking adversity to atypical amygdala function and development^63^. Although the amygdala is classically described as a central node of the fear network, this region probably supports both ABT and ABP^3^, with more recent studies demonstrating a large overlap of fear and reward networks (e.g.^64,65^). Investigating links between development in these networks and a relation between affect-biased attention and socioemotional functioning over time will be key in future studies.

Strengths of the current study include the use of ecological dynamic stimuli and investigation of both ABT and ABP links to socioemotional behaviour. In addition, the use of a macaque model allowed for very well-controlled consideration of early environmental effects. In human research, it often difficult to distinguish effects of the early social environment from other factors such as physical neglect, but results here provide support for the effects of very early social deprivation specifically on affect-biased attention in the pre-adolescent period. However, there are some limitations to this study that should be noted. First, the modest sample size could represent a limitation, and it is important that the results are confirmed in larger samples Second, these results do not speak to the issue of trait versus state anxiety, both of which have been linked to affect-biased attention (see^11^), which will require the measurement of anxiety-like behaviour in different contexts across an extended time scale. Third, atypical ABP may also play a role in adverse behavioural outcomes in human children (e.g.^66^), therefore prospective macaque research should consider this also.

There are three additional points concerning the group differences revealed here that need to be considered. Firstly, although animals in the mother-reared group remained in their natal group for the first eight months of life, they were also removed from their natal group post-weaning. Therefore, it is possible that our results were affected by ‘earlier vs later’ maternal separation. Future studies would ideally include another group of animals that were not removed from their natal social group at any point. However, our results do demonstrate clear differences between the two rearing groups, and are in keeping with previous findings concerning development of affect biased attention in individuals who have been exposed to early psychosocial adversity versus not (e.g.^20,25^). Behaviour in our peer-reared group (e.g. increased anxiety-like behaviour) does suggest that their risk for such long-term poor outcomes is greater than in the mother-reared group, perhaps highlighting the first months of life as a sensitive period for the impact of social adversity on certain aspects of socioemotional development. Secondly, peer-reared animals often obtain a lower social rank than mother-reared animals (e.g.^67^), and hypothetically, exposure to more threat in the environment as a result of low rank may have a direct effect on anxiety, or indirectly via effects on attention biases. In our sample, peer-reared animals did tend to be lower-ranking (see SI), though the inclusion of rank as a covariate in our models controlled for this factor. This suggests that early social adversity affects rank and gaze bias/behaviour independently, but is still possible that rank moderates the mediating effects of attention biases. Indeed, some previous work with adult macaques has found that social dominance can influence attention towards social stimuli (e.g.^68^). This possibility is an interesting avenue for future longitudinal work with larger samples, especially in the case of natural variation in early social experience. And thirdly, while social grooming is often used as a measure of prosocial engagement in macaques, grooming is a complex behaviour with context-dependent effects of hormonal and neural responses in NHPs^69,70^. Elucidating these context-dependent effects such as the relative rank of the actor and receiver, and their relationship to affect-biased attention will be important in future research.

To conclude, this study demonstrates that juvenile, pre-adolescent macaques are biased towards looking at both threatening and affiliative dynamic facial gestures, but that the degree of attention bias is influenced by early social adversity. Furthermore, links between ABT and anxiety in the context of early adversity, and an absence of ABP links with social engagement, suggests that affect-biased attention could play an important role in rhesus macaque development. Longitudinal research concerning the mechanisms underlying these relations is now required to determine the factors conferring greatest risk for anxiety, as well as those implicated in positive social outcomes and resilience.

## Supplementary Information

Supplementary Information.
