# Author Correction: Early social adversity modulates the relation between attention biases and socioemotional behaviour in juvenile macaques

**DOI:** 10.1038/s41598-025-28538-w

**Published:** 2025-12-08

**Authors:** Holly Rayson, Alice Massera, Mauro Belluardo, Suliann Ben Hamed, Pier Francesco Ferrari

**Affiliations:** 1 Institut des Sciences Cognitives Marc Jeannerod, Centre National de la Recherche Scientifique, Université Claude Bernard Lyon 1, Bron, France; 2 Unit of Neuroscience, Department of Medicine and Surgery, University of Parma, Parma, Italy

Correction to: *Scientific Reports* 10.1038/s41598-021-00620-z, published online 04 November 2021

The original version of the Article contained errors.

After publication, the Authors noted a computing error in the behavioural preprocessing pipeline that affected how summary statistics for behavioural indices (anxiety frequency, duration of social engagement, and frequency of social engagement) were calculated. Namely, some grooming actions were counted twice, while behaviours that occurred at the start or end of the 5-minute coded segment were excluded. The Authors now fixed the behavioural preprocessing pipeline, so that it correctly calculates the anxiety-related behaviour and grooming summary statistics. The preprocessing script has been publicly archived on GitHub (https://github.com/hrayson/attentionbiasmacaque).

As a result, In the Methods section, under the subheading ‘Data analysis’,

“To calculate attention bias to threat (ABT), the proportion of time looking at the neutral and threat stimuli (out of total time looking onscreen) was calculated separately for each Neutral-Threat trial, with the neutral proportion then subtracted from the threat proportion.”

now reads:

“Preprocessing and analyses pipeline scripts are available on GitHub (https://github.com/hrayson/attentionbiasmacaque). To calculate attention bias to threat (ABT), the proportion of time looking at the neutral and threat stimuli (out of total time looking onscreen) was calculated separately for each Neutral-Threat trial, with the neutral proportion then subtracted from the threat proportion.”

Additionally, in the Results section, under the subheading ‘Relation between attention biases and socioemotional behaviour’,

“We found a group × ABT interaction [*χ*^2^(1) = 6.256, *p* = 0.012, mother-reared effect size = − 0.193, peer-reared effect sized = 0.506] (Fig. 3), with greater ABT related to more frequent anxiety-like behavior in the peer-reared group [*z* = 2.261, *p* = 0.024]. There was no significant main effect of ABP or the relation between ABP and frequency of anxious behaviour in either rearing group (both *p* > 0.458).”

now reads:

“We found a group × ABT interaction [*χ*^2^(1) = 5.976, *p* = 0.015, mother-reared effect size = − 0.178, peer-reared effect sized = 0.504] (Fig. 3), with greater ABT related to more frequent anxiety-like behavior in the peer-reared group [*z* = 2.256, *p* = 0.024]. There was no significant main effect of ABP or the relation between ABP and frequency of anxious behaviour in either rearing group (both *p* > 0.411).”

and

“We then went on to investigate relations between attention biases and the frequency and duration of social engagement. There were significant main effects of ABP [*χ*^2^(1) = 10.064, *p* = 0.002, effect size = 1.070], group [*χ*^2^(1) = 7.445, *p* = 0.006, effect size (mother-peer) = − 1.199], and an ABP × group interaction [*χ*^2^(1) = 6.194, *p* = 0.013, mother-reared effect size = 1.931, peer-reared effect size = 0.211] for social engagement frequency (Fig. 4a), with greater ABP related to more frequent social engagement in the mother-reared group [*z* = 3.172, *p* = 0.002]. For social engagement duration, we found significant main effects of both ABP [*χ*^2^(1) = 18.397, *p* ≤ 0.0001, effect size = 2.56] and group [*χ*^2^(1) = 12.746, *p* ≤ 0.001, effect size (mother-peer) = − 60.590], and an ABP × group interaction [*χ*^2^(1) = 9.793, *p* = 0.002, mother-reared effect size = 4.456, peer-reared effect size = 0.665]; greater ABP was related to a longer duration of social engagement [*z* = 4.289, *p* < 0.0001 in the mother-reared group (Fig. 4b). There were no significant main effects of ABT or relations between ABT and frequency or duration of social engagement (all *p* > 0.236).”

now reads:

“We then went on to investigate relations between attention biases and the frequency and duration of social engagement. There were significant main effects of ABP [*χ*^2^(1) = 10.788, *p* = 0.001], group [*χ*^2^(1) = 5.169, *p* = 0.023], and an ABP × group interaction [*χ*^2^(1) = 7.730, *p* = 0.005, mother-reared effect size = 2.278, peer-reared effect size = 0.004] for social engagement frequency (Fig. 4a), with greater ABP related to more frequent social engagement in the mother-reared group [*z* = 3.284, *p* = 0.001]. For social engagement duration, we found significant main effects of both ABP [*χ*^2^(1) = 16.547, *p* ≤ 0.0001] and group [*χ*^2^(1) = 5.891, *p* ≤ 0.015], and an ABP × group interaction [*χ*^2^(1) = 11.481, *p* = 0.001, mother-reared effect size = 3.784, peer-reared effect size = 0.064]; greater ABP was related to a longer duration of social engagement [*z* = 4.068, *p* < 0.0001 in the mother-reared group] (Fig. 4b). There were no significant main effects of ABT or relations between ABT and frequency or duration of social engagement (all *p* > 0.528).”

Furthermore, the original version of this Article contained errors in Table 1, where the values in the section titled “Socioemotional Behavior” were reported incorrectly. The correct and incorrect sections appear below.

Incorrect:

**Table:** 

*Socioemotional behaviour*		
Anxiety frequency	1.561 (0.743)	2.383 (1.114)
Social groom frequency	1.106 (0.987)	0.983 (0.713)
Social groom duration (s)	50.606 (48.466)	37.117 (31.096)

Correct:

**Table:** 

*Socioemotional behaviour*		
Anxiety frequency	1.515 (0.701)	2.350 (1.093)
Social groom frequency	1.485 (1.599)	1.250 (0.934)
Social groom duration (s)	42.909 (45.546)	24.100 (21.869)

In addition, the original version of this Article contained an error in Figure 3, where the values were reported incorrectly. The incorrect Figure 3 along with its caption is provided below.

**Fig. 3:**
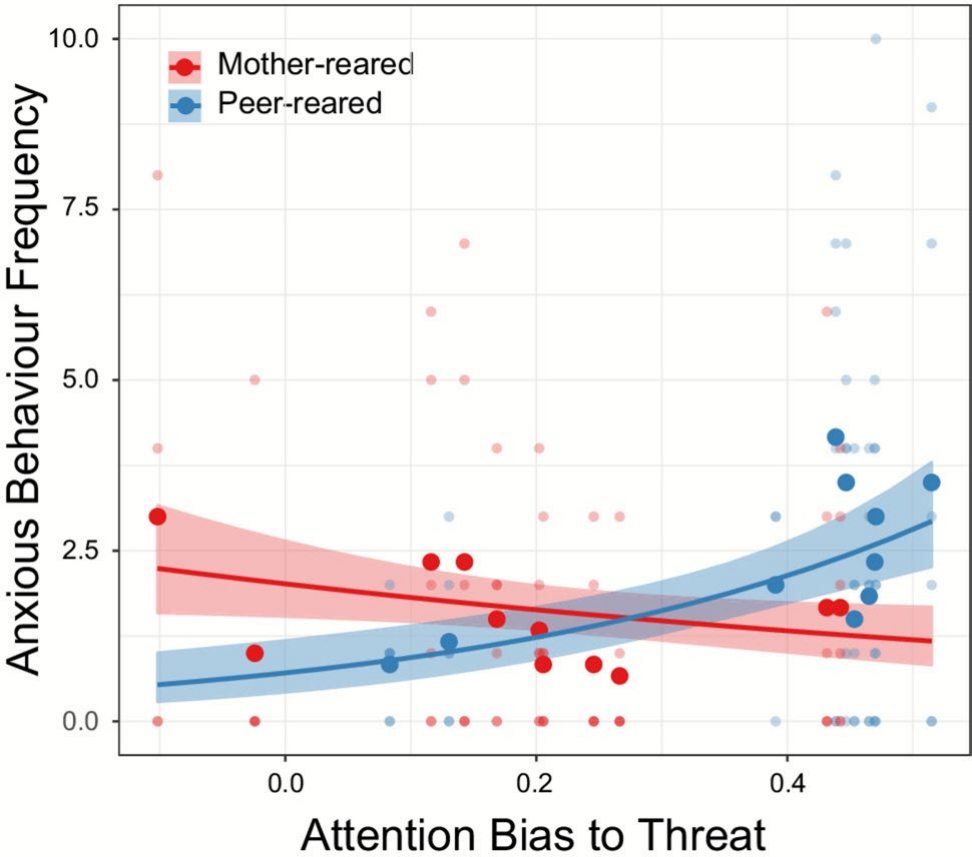
The relation between attention bias to threat (ABT) and frequency of anxiety-like behaviour in the mother-reared (red) and peer-reared (blue) group. Each light-coloured dot represents an individual behavioural observation session, large dark-coloured dots represent the subject mean, dark lines indicate the model fit, and shaded regions around the lines denote + or − SE.

Also, the original version of this Article contained an error in Figure 4, where the values were reported incorrectly. The incorrect Figure 4 along with its caption is provided below.

**Fig. 4:**
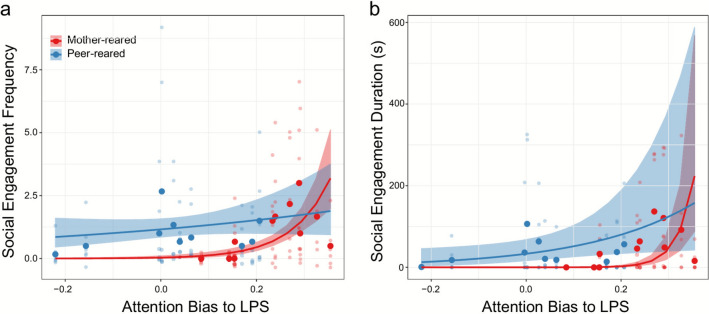
The relations between attention bias to LPS (i.e. to positive stimuli; ABP) and social engagement in the mother–reared (red) and peer-reared (blue) group. Each light-coloured dot represents an individual behavioural observation session, large dark-coloured dots represent the subject mean, dark lines indicate the model fit, and shaded regions around the lines denote + or − SE.

Finally, in the original article the term “peer-reared” was used to refer to animals that were not mother-reared and raised in early life without consistent maternal care. However, as per Reference 48, this group includes two subtypes: surrogate-reared animals, which were housed individually (N = 6); and peer-reared animals, which were raised in contact with one or more conspecifics from one month after birth within the nursery setting (N = 4). After publication, it was brought to Authors’ attention that it was not explicit in the text that the two subtypes were included as part of the peer-reared group. To clarify and validate the decision to group the two subtypes, the Authors now repeated all analyses using a three-level rearing variable: mother-reared, peer-reared, and surrogate-reared. These results are presented in the Supplementary Information, in the “Three-Group Comparison of Rearing Conditions” section. The results do not differ meaningfully from those originally reported and support the decision to group the two subtypes.

Consequently, the Supplementary Information file published with this Article has been updated. The original Supplementary Information file is provided below.

The original Article and accompanying Supplementary Information file have been corrected.

## Supplementary Information

Supplementary Information.

