# Supplementary material for: Author Correction: Early social adversity modulates the relation between attention biases and socioemotional behaviour in juvenile macaques
Source: Sci Rep. 2025 Dec 8;15:43260. doi: 10.1038/s41598-025-28538-w (PMC12686509; doi:10.1038/s41598-025-28538-w)
Supplement: Supplementary file 1 — Supplementary Information. [file 41598_2025_28538_MOESM1_ESM.docx]

**EARLY SOCIAL ADVERSITY MODULATES THE RELATION BETWEEN ATTENTION BIASES AND SOCIOEMOTIONAL BEHAVIOUR IN JUVENILE MACAQUES**

***Holly Rayson^1^, Alice Massera^1^, Mauro Belluardo^2^, Suliann Ben Hamed^1^, & Pier Francesco Ferrari^1, 2^**

^1^ Institut des Sciences Cognitives, Centre National de la Recherche Scientifique, Université Claude Bernard Lyon 1, Bron, France

^2^ Unit of Neuroscience, Department of Medicine and Surgery, University of Parma, Parma, Italy

***Corresponding author:** h.rayson@isc.cnrs.fr

**Address:** Institut des Sciences Cognitives Marc Jeannerod, CNRS, 67 Boulevard Pinel, 69675, Bron, France

**Supplementary Information**

**Methods**

***Subjects***

Peer-reared animals were raised from birth in a nursery with access to same-aged peers; this peer-rearing protocol was based on that of Shannon et al. (1998). At eight months postpartum, mother-reared and peer-reared subjects were placed into a single social group. Animals were relocated to their current location at two years of age and are housed altogether. Animals live in semi free-ranging conditions, with access to both indoor and outdoor areas. As part of a wider longitudinal study, animals are temporarily relocated once a year to a nearby location in groups of 5-6 animals. All groups contain a balanced mix of mother-reared and peer-reared animals, with groups housed in an indoor enclosure. This enclosure is enriched to allow for meaningful activities and expression of the animals’ full behavioural repertoire. Assessments for the current study were conducted a minimum of 10 days after this relocation.

***Facial gesture stimuli***

To calculate brightness, contrast, spatial frequency, and overall movement, video frames were first converted to greyscale by averaging over the RGB colour dimension. Brightness was computed as the normalized average of all pixel values, and contrast as the standard deviation. Spatial frequency was computed using a two-dimensional Fourier transform, which was then averaged over the azimuth to create a one-dimensional vector of power at each spatial frequency. Motion was computed using the Farneback method (Farnebäck, 2003) for optical flow, using a classical pyramid image scale with 3 levels, an averaging window size of 15 pixels, 3 iterations per pyramid, a pixel neighborhood size of 5, and a Gaussian with SD equal to 1.2 for derivative smoothing; movement was then converted to pixels per second.

The values averaged over all frames from each video for each facial gesture type were as follows: a) Lip-smacking (LPS), brightness = 0.337, contrast = 0.168, motion = 52.13 pixels per second; b) Neutral, brightness = 0.335, contrast = 0.2, motion = 41.86 pixels per second; and c) Threat, brightness = 0.34, contrast = 0.2, motion = 42.81 pixels per second. Brightness, contrast, spatial frequency, and motion are illustrated in Figures S1-S4.


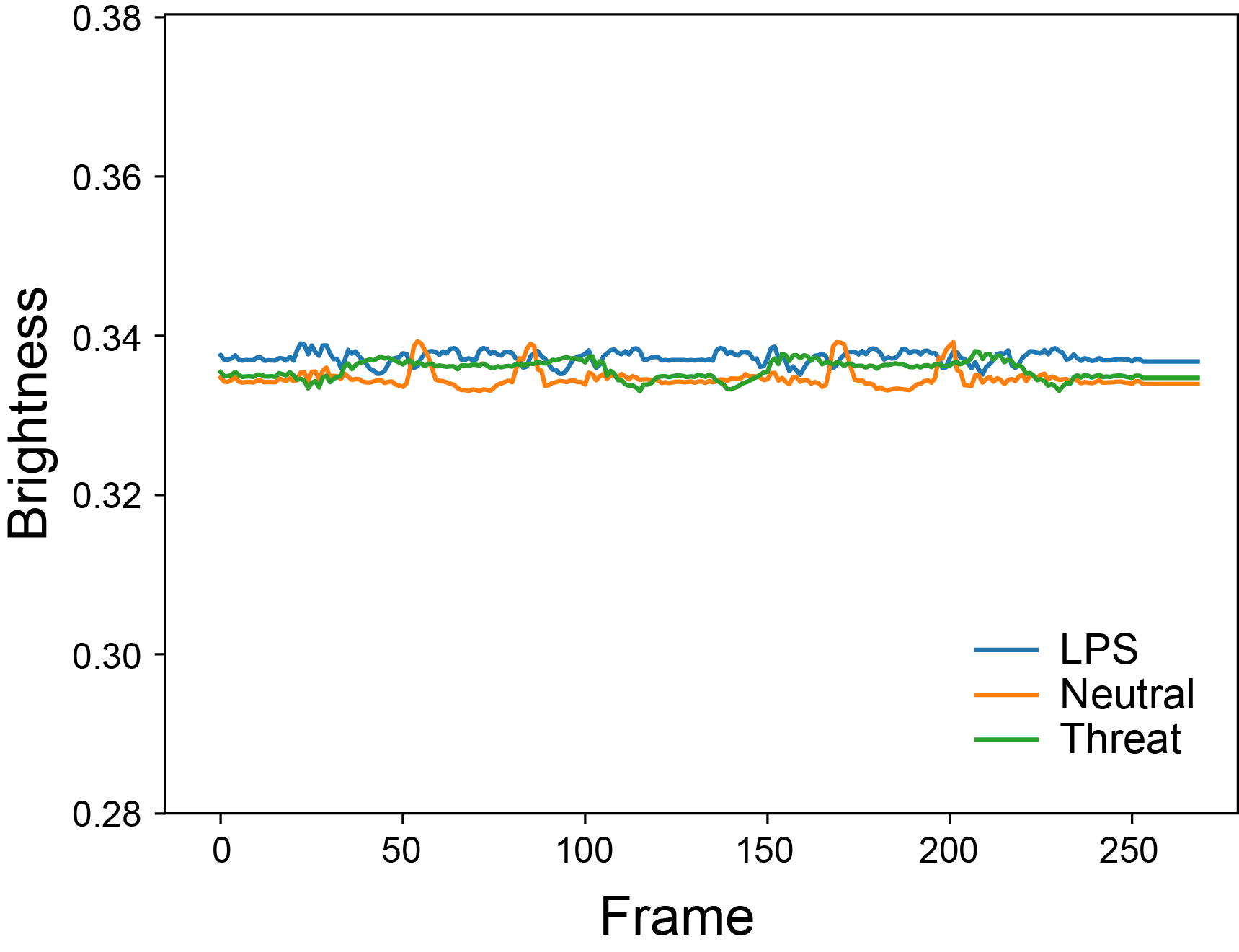

***Figure S1:*** *Brightness for LPS (blue), neutral (orange), and threat (green) videos computed as the normalized average of all pixel values.*


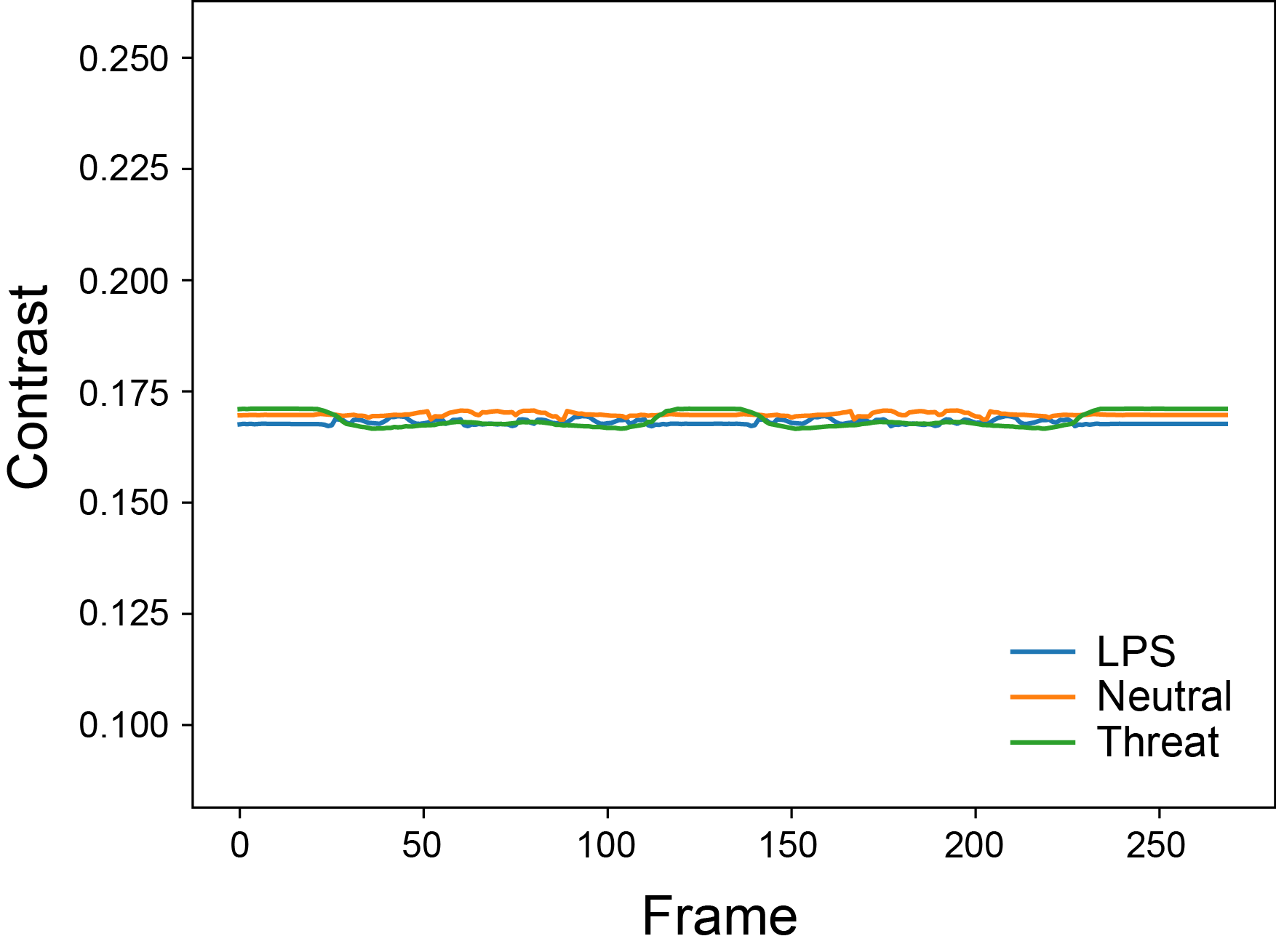


**Figure S2:** Contrast for LPS (blue), neutral (orange), and threat (green) videos computed as the standard deviation of all pixel values.


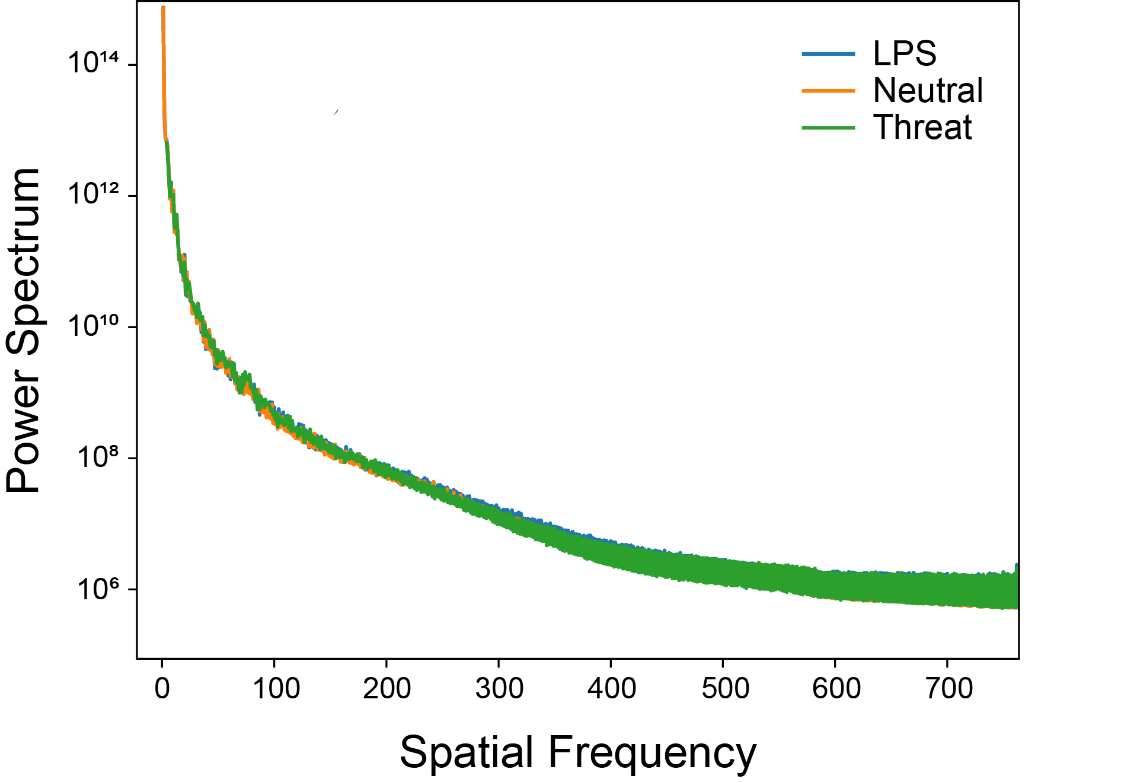


**Figure S3:** Spatial power spectrum for LPS (blue), neutral (orange), and threat (green) videos computed using a two-dimensional Fourier transform, averaged over the azimuth to create a one-dimensional vector of power at each spatial frequency.


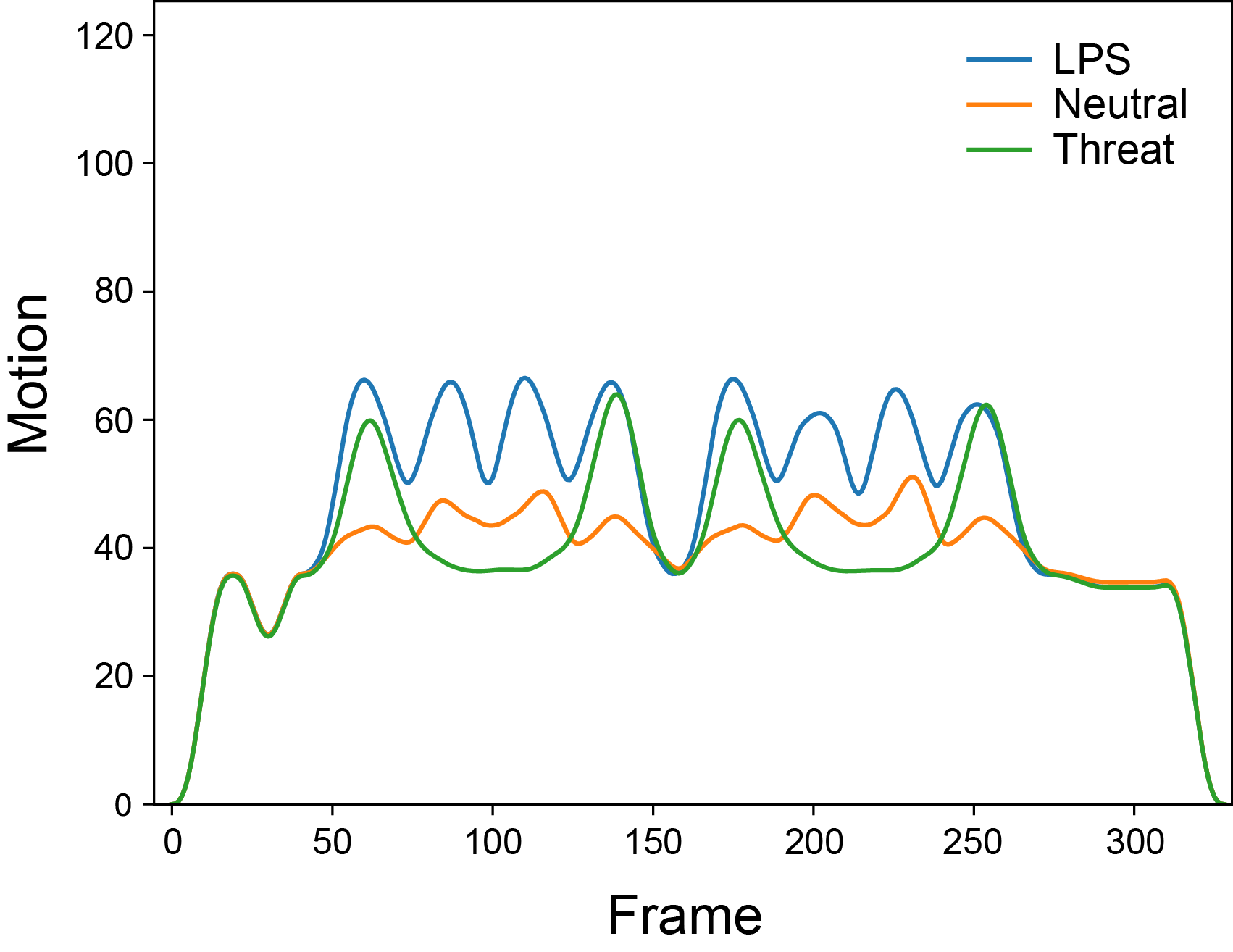


**Figure S4:** Motion (pixels per second) for LPS (blue), neutral (orange), and threat (green) videos computed using the Farneback method for optical flow.

**Data analysis**

R v3.6.3 (R Core Team, 2020) and the lme4 v1.1.21 (Bates et al., 2015), nlme v3.1.144 (Pinheiro et al., 2020), lsmeans v2.30.0 (Lenth, 2016), the glmmADMB v0.8.3.3 (Fournier et al., 2012; Skaug et al., 2016), and car v3.0.6 (Fox & Weisberg, 2019) packages were utilized to conduct these analyses.

**Results**

***Gaze bias task: Trial exclusion in the different conditions***

A generalized linear model with a Poisson distribution and logit link function was run to confirm that the number of gaze bias trials did not differ between groups or conditions after trail rejection. This revealed no effect of group, condition, or their interaction on the number of trials. Mean trials (out of the 5 ABT trials and 5 ABP trials per subject) included for mother-reared animals were as follows: ABT, M = 4.818, SD = 0.405, range 4-5; ABP, M = 4.909, SD = 0.302, range 4-5: Mean trials included for peer-reared animals were as follows: ABT, M = 4.7, SD = 0.483, range 4-5; ABP, M = 4.6, SD = 0.699, range 3-5.

***Social rank calculation***

Rank of individual animals was established via Elo-ratings (Sanchez-Tojar et al., 2018; R packages ‘EloRating’ v 0.46.11; Neumann & Kulik, 2020; ‘aniDom’ v0.1.5; Farine & Sanchez-Tojar, 2021), with winners and losers of agonistic interactions, classified as aggressive or submissive, identified from 60 minutes of behavioural observation per animal (four observations per week (two morning, two afternoon) for three weeks). Coded behaviours included displacement, threat gestures, aggression, fear grimace, and present genitals (Dettmer et al., 2016; Wooddall et al., 2017). There were a total of 198 interactions, M=9.41 and SD=1.81 per group. As the hierarchies were highly stable over the course of the observation period (S index: 0.95 – 0.99; M=0.97; SD=0.02), we used randomized Elo-ratings (over 100 temporally shuffled randomizations). Animals in the peer-reared (M=-201.25, SD=180.21) group attained a lower rank on average than the mother-reared (M=182.96, SD=141.87) group [t(17.11)=5.39, p<0.001].

***Influence of sex***

To control for a potential influence of sex on our results, we also ran all analyses reported in the main text with sex included as a covariate. All animals were included in these analyses; 11 mother-reared (six female) and 10 peer-reared (five female). The pattern of results revealed were identical to those reported in the main manuscript, suggesting that differences between groups remain even when accounting for sex.

**References**

Bates, D., Maechler, M., Bolker, B., & Walker, S. (2015). Fitting Linear Mixed-Effects Models Using lme4. *Journal of Statistical Software, 67*(1), 1-48. doi:10.18637/jss.v067.i01.

Dettmer, A. M., Wooddell, L. J., Rosenberg, K. L., Kaburu, S. K. K, Novak, M. A., Meyer, J. S., & Suomi, S. J. (2016). Associations between early life experience, chronic HPA axis activity, and adult social rank in rhesus monkeys. *Social Neuroscience, 12*(1), 92–101.

Farine, D. R, & Sanchez-Tojar, A. (2021). aniDom: Inferring Dominance Hierarchies and Estimating Uncertainty. R package version 0.1.5. https://CRAN.R-project.org/package=aniDom.

Fournier, D. A., Skaug, H. J., Ancheta, J., Ianelli, J., Magnusson, A., Maunder, M. N., ... & Sibert, J. (2012). AD Model Builder: using automatic differentiation for statistical inference of highly parameterized complex nonlinear models. *Optimization Methods and Software*, *27*(2), 233-249.

Lenth, R.V. (2016). Least-Squares Means: The R Package lsmeans. *Journal of Statistical Software, 69*(1), 1-33. doi:10.18637/jss.v069.i01

Neumann, J. & Kulik, L. (2020). EloRating: Animal Dominance Hierarchies by Elo Rating. R package version 0.46.11, URL: https://CRAN.R-project.org/package=EloRating.

Pinheiro, J., Bates, D., DebRoy, S., Sarkar, D., R Core Team. (2020). _nlme: Linear and Nonlinear Mixed Effects Models_. R package version 3.1-144, URL: https://CRAN.R-project.org/package=nlme.

R Core Team. (2020). R: A language and environment for statistical computing. R Foundation for Statistical Computing, Vienna, Austria. URL https://www.R-project.org/.

Sánchez‐Tójar, A., Schroeder, J., & Farine, D. R. (2018). A practical guide for inferring reliable dominance hierarchies and estimating their uncertainty. *Journal of Animal Ecology*, *87*(3), 594-608.

Skaug, H., Fournier, D., Bolker, B., Magnusson, A., & Nielsen, A. (2016). Generalized Linear Mixed Models using ‘AD Model Builder’. R package version 0.8.3.3.

Wooddell, L. J., Kaburu, S., Murphy, A. M., Suomi, S. J., & Dettmer, A. M. (2017). Rank acquisition in rhesus macaque yearlings following permanent maternal separation: The importance of the social and physical environment. *Developmental psychobiology*, *59*(7), 863–875. https://doi.org/10.1002/dev.21555
